# Clinical and radiological characteristics of novel subtypes of end-stage knee osteoarthritis based on joint space loss patterns in standing extended view and fixed flexion view

**DOI:** 10.1186/s12891-025-08943-y

**Published:** 2025-07-22

**Authors:** Woo-Suk Lee, Tae Hyung Kim, Hyuck Min Kwon, Jun Young Park, Kwan Kyu Park, Byung-Woo Cho

**Affiliations:** 1 Department of Orthopaedic Surgery, Gangnam Severance Hospital, Yonsei University College of Medicine, 211 Eonju-ro, Gangnam-gu, Seoul, 06273 Republic of Korea; 2 Department of Orthopaedic Surgery, Severance Hospital, Yonsei University College of Medicine, 501 Yonseiro, Seodaemungu, Seoul, 03722 Republic of Korea; 3 Department of Orthopaedic Surgery, Yongin Severance Hospital, Yonsei University College of Medicine, 363, Dongbaekjukjeon-daero, Giheung-gu, Yongin-si, 16995 Gyeonggi-do Republic of Korea

**Keywords:** Knee osteoarthritis, Standing extended view, Fixed flexion view, Posterior tibial slope, Total knee arthroplasty

## Abstract

**Background:**

This study aimed to classify end-stage knee osteoarthritis (KOA) based on the pattern of joint space loss in standing extended view (SEV) and fixed flexion view (FFV) and to investigate clinical and radiological differences.

**Methods:**

A total of 459 knees from 300 patients with Kellgren-Lawrence grade 4 KOA were retrospectively analyzed. The knees were divided into three groups based on the pattern of joint space loss in SEV and FFV: group 1 (all loss) with joint space loss in both SEV and FFV, group 2 (flexion loss) with joint space loss only in FFV, and group 3 (extension loss) with joint space loss only in SEV. The primary endpoints were clinical and radiological parameters, while the secondary endpoints included intraoperative measurements and the survival rate until total knee arthroplasty (TKA).

**Results:**

A total of 459 knees from 300 patients were included. Among the participants, there were 77 men (25.7%) (average age of 72.21 ± 7.35 years), and 223 women (74.3%) (average age of 72.75 ± 6.56 years) (*p* = 0.546). Compared to group 2, group 1 showed a larger hip-knee-ankle angle (9.8 ± 7.0° and 6.3 ± 5.0°, *p* < 0.001), higher VAS (6.3 ± 2.4 and 4.6 ± 2.5, *p* < 0.001), shorter time to surgery (7.1 ± 7.7 months and 11.0 ± 8.7 months, *p* < 0.001), smaller full flexion angle (114.3 ± 13.4° and 121.2 ± 11.9°, *p* = 0.001), and a higher total knee arthroplasty rate (76% and 57.2%, *p* < 0.001). Group 3 showed a larger flexion contracture angle compared to group 2 (10.00 ± 9.6° and 5.3 ± 5.4°, *p* = 0.032). The posterior tibial slope (PTS) was largest in group 2 (11.3 ± 3.3°), followed by group 1 (8.1 ± 3.3°), and smallest in group 3 (5.4 ± 2.7°) (both *p* < 0.001, respectively). There were no statistical differences in the intra-operative measurements. TKA was performed on 259 knees (64.3%), and the survival rates at 1 year were 48.1% for group 2, 29.2% for group 3, and 26.7% for group 1 (log-rank test, *p* < 0.001).

**Conclusions:**

This study demonstrates that radiological and clinical differences exist within end-stage KOA based on joint space loss patterns. Additionally, our findings suggest that a larger PTS may be associated with less symptom severity in advanced KOA, contrary to its currently recognized negative effects. These findings may be beneficial for developing patient-specific treatment plans.

**Level of evidence:**

Retrospective cohort study, Level III

**Supplementary Information:**

The online version contains supplementary material available at 10.1186/s12891-025-08943-y.

## Introduction

Given that the severity of knee osteoarthritis (KOA) correlates with symptoms [1] and determines diverse treatment strategies [2], accurately evaluating its severity is deemed essential in managing KOA patients. Among various imaging modalities used for evaluation, simple radiographs are commonly the first choice due to their simplicity, quickness, and ability to reflect weight-bearing conditions [3]. There are various classifications utilizing simple radiographs for evaluating KOA, and most scales define end-stage osteoarthritis (OA) as the presence of severe joint space narrowing [4].

To evaluate OA of the tibiofemoral joint using simple radiographs, it is necessary to obtain not only the weight-bearing standing extended view (SEV) but also the fixed flexion view (FFV), which reflects the posterior aspect of the joint. The FFV is a radiographic examination where the patient bends their knee approximately 20–45 degrees while standing [5], and it is known to be advantageous for evaluating OA of the tibiofemoral joint compared to simple standing radiographs [5, 6]. The femoral condyles roll during early flexion, resulting in the joint space observed in FFV being more posterior compared to the SEV [7]. Therefore, depending on the type of KOA, variations in joint space between SEV and FFV can occur. In some patients, joint space loss is observed only in SEV or FFV, while others show joint space loss in both SEV and FFV. Theoretically, these subtypes of KOA may differ in symptoms or underlying mechanisms, but there is a lack of studies analyzing these characteristics. Most previous radiographic studies evaluating KOA have focused on the absolute value of joint space width, but the relationship between joint space loss patterns in different positions (extension vs. flexion) and patient symptoms has not been elucidated. Therefore, this study aimed to classify end-stage KOA patients based on joint space loss in SEV and FFV and elucidate their clinical and radiological differences. We hypothesized that patients with different patterns of joint space loss would differ in anatomical structures, clinical pain scores, and time to surgical intervention.

## Patients/Methods

### Subject recruitment & data collection

This retrospective study was approved by the Institutional Review Board (IRB) of our institution. This study enrolled patients with end-stage KOA with Kellgren-Lawrence (KL) grade 4, who first visited the outpatient orthopedic clinic of our institution from January to December 2022 and had all the required radiographic images available. Exclusion criteria were as follows: (1) patients with end-stage KOA limited to the patellofemoral joint; (2) history of previous surgeries, such as ligament reconstruction or osteotomy; (3) patients with fractures, including subchondral insufficiency fractures; (4) patients with pigmented villonodular synovitis; (5) patients with rheumatoid arthritis; (6) patients with concomitant motor weakness; (7) patients who were lost to follow-up.

Patient data were collected from hospital electronic medical record (EMR) system. The patient’s age, sex, body mass index (BMI), range of motion (ROM), affected side, duration of patient-reported symptoms, history of previous partial meniscectomy, visual analogue scale (VAS) pain score during daily activities at the initial outpatient visit, whether total knee arthroplasty (TKA) was performed, and the time from the first outpatient visit to surgery were recorded. ROM was measured by an orthopedic surgeon using a goniometer, and VAS scores were recorded separately for each knee on a scale of 0 to 10. Follow-up for survival analysis was conducted until January 2024.

### Radiologic assessments

**Fig. 1:**
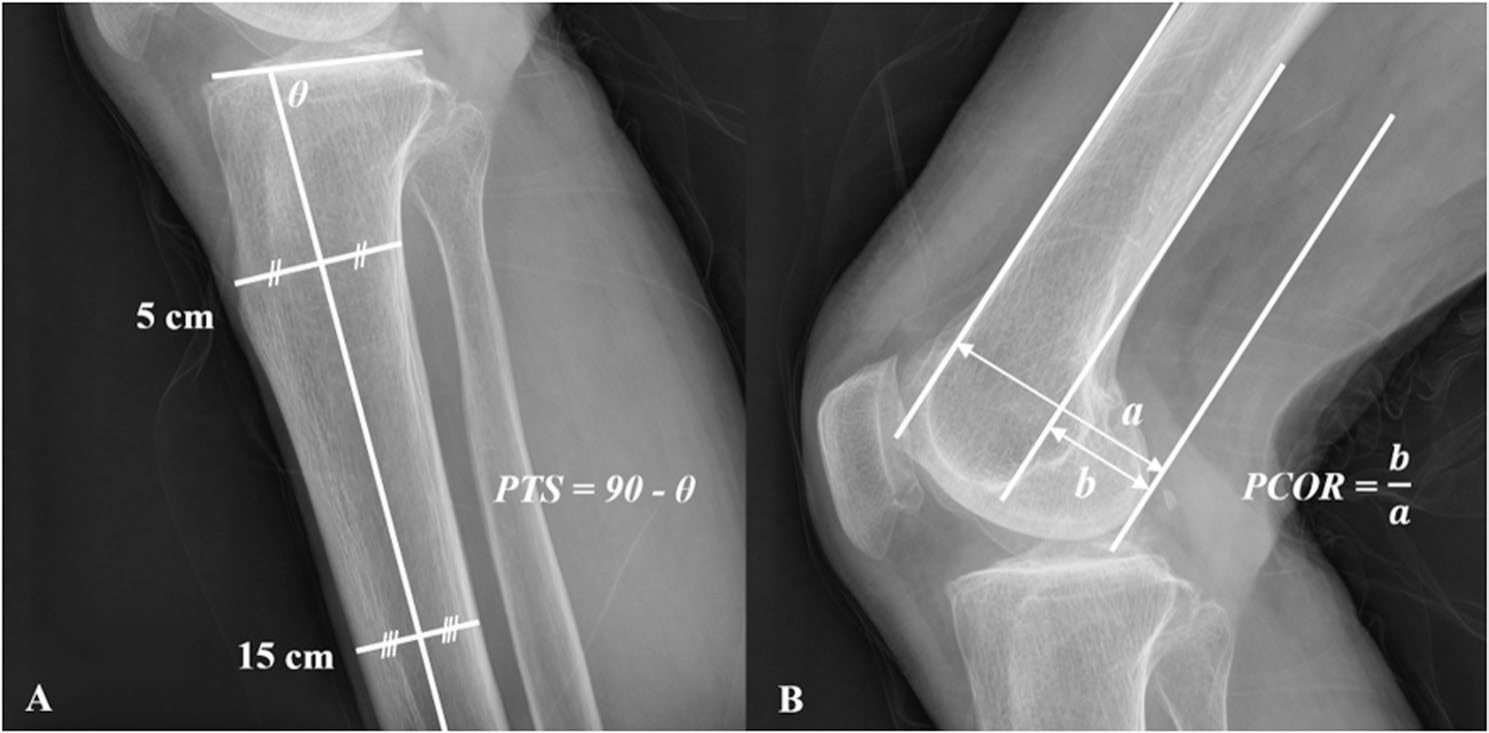
Definitions of (**A**) Posterior Tibial Slope (PTS) and (**B**) Posterior Condylar Offset Ratio (PCOR)

Radiological measurements were conducted using simple radiographs obtained at the first outpatient visit, and were blindly assessed by two coauthors. The SEV was obtained with the patient standing in a position that allowed the patella to face forward. The FFV was acquired with the patient in an upright position, flexing the knee to approximately 45 degrees, while the X-ray beam was angled 10 degrees cranially. The posterior tibial slope (PTS) was measured as described in previous literature, using the proximal anatomical axis defined by the midpoint between the 5 cm and 15 cm points on the medial tibial plateau [8] (Fig. 1A). The posterior condylar offset ratio (PCOR) was also defined according to previous literature, using tangents of the femoral diaphysis cortex and the posterior condylar margin on a true lateral view [9] (Fig. 1B). The hip-knee-ankle angle (HKA) was measured on standing scanograms using the centers of each joint [10]. HKA angles of valgus knees were recorded as negative values.

**Fig. 2:**
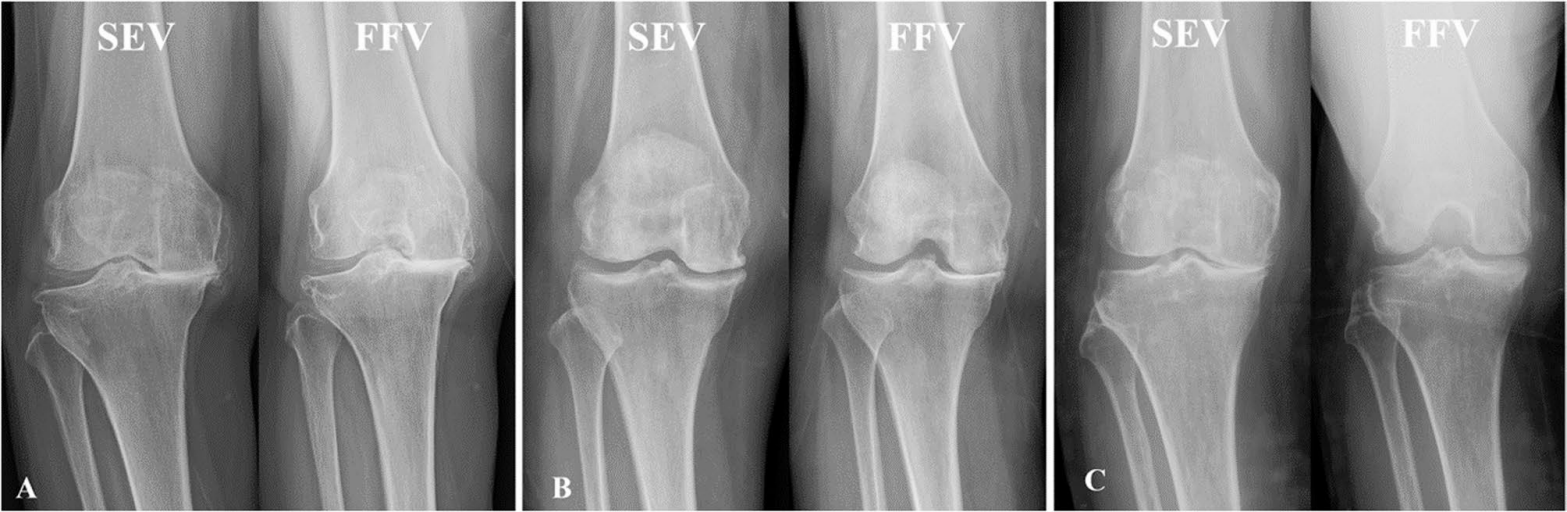
Classification based on patterns on joint space loss. (**A**) group 1 (all loss) with joint space loss in both standing extended view (SEV) and fixed flexion view (FFV) (**B**) group 2 (flexion loss) with joint space loss only in FFV, (**C**) group 3 (extension loss) with joint space loss only in SEV

In all knees, the involved compartment was identified, and the joint space in SEV and FFV was evaluated in three categories: spared, narrowing, and loss. “Spared” was defined as no difference compared to the uninvolved compartment; “narrowing” was defined as reduced space with some joint space remaining; and “loss” was defined as no joint space. Based on these categories, knees were divided into three groups (Fig. 2). Group 1 (all loss) included knees that showed joint space loss in both SEV and FFV (Fig. 2A). Group 2 (flexion loss) included knees with spared or narrowed joint space in SEV but loss in FFV (Fig. 2B). Group 3 (extension loss) included knees with joint space loss in SEV but spared or narrowed joint space in FFV (Fig. 2C). Patient data and various measurements were compared and analyzed among these three groups.

Intra- and inter-observer reliabilities for radiographic parameters were as follows: 0.967 and 0.933 for PTS, 0.877 and 0.861 for PCOR, 0.983 and 0.965 for HKAs, and 0.923 and 0.851 for the KOA subtype.

### Intra-operative measurements

The indication for TKA was determined based on persistent pain and functional limitation despite conservative treatment, and the decision to undergo surgery was ultimately made by the patient in consultation with the orthopedic surgeon. All surgeries were performed with a posterior stabilized (PS) type TKA by two orthopedic surgeons, WSL (over 25 years’ experience) and BWC (over 10 years’ experience). All procedures were conducted using a mechanically aligned technique, and the rotation of the femoral component was determined using the gap-balancing method. The proximal tibial resection was initiated using a stylus, with a 2 mm resection referenced from the defective tibial plateau. Posterior femoral condylar resection was performed after determining the femoral component size using the anterior referencing technique. For patients who underwent TKA, intraoperative gaps and space measurements were performed using the offset type tensor FuZion (Zimmer Biomet, Warsaw, IN) after distal femoral and proximal tibial resections [11]. Following meniscectomy and deep medial collateral ligament release, the device applied 40 lbf of distraction force, and the distance and angle between the resected bone surfaces were measured accordingly.

### Statistical analysis

The normality of the variables was assessed using the Shapiro–Wilk test. One-way analysis of variance (ANOVA) and the Kruskal-Wallis test were used to compare continuous variables among the three groups. Post-hoc analysis was performed using Bonferroni’s method. The Chi-square test and Fisher’s exact test were employed to compare categorical variables among the three groups. Kaplan–Meier survival analysis was performed using TKA as the endpoint. Statistical analysis was performed using SPSS software for Windows (v.28.0; SPSS Inc., Chicago, IL, USA) and R (v.4.4.3; RStudio, PBC, Boston, MA, USA). Post hoc power analysis based on the primary variable, PTS, using an alpha error of 0.05, demonstrated a statistical power of 1.00. The level of statistical significance was set at *p* < 0.05. Intra- and inter-observer reliabilities for all measurements were assessed by two coauthors, each with over 10 years of experience, using intraclass correlation coefficients and Cohen’s kappa coefficients.

## Results

**Fig. 3:**
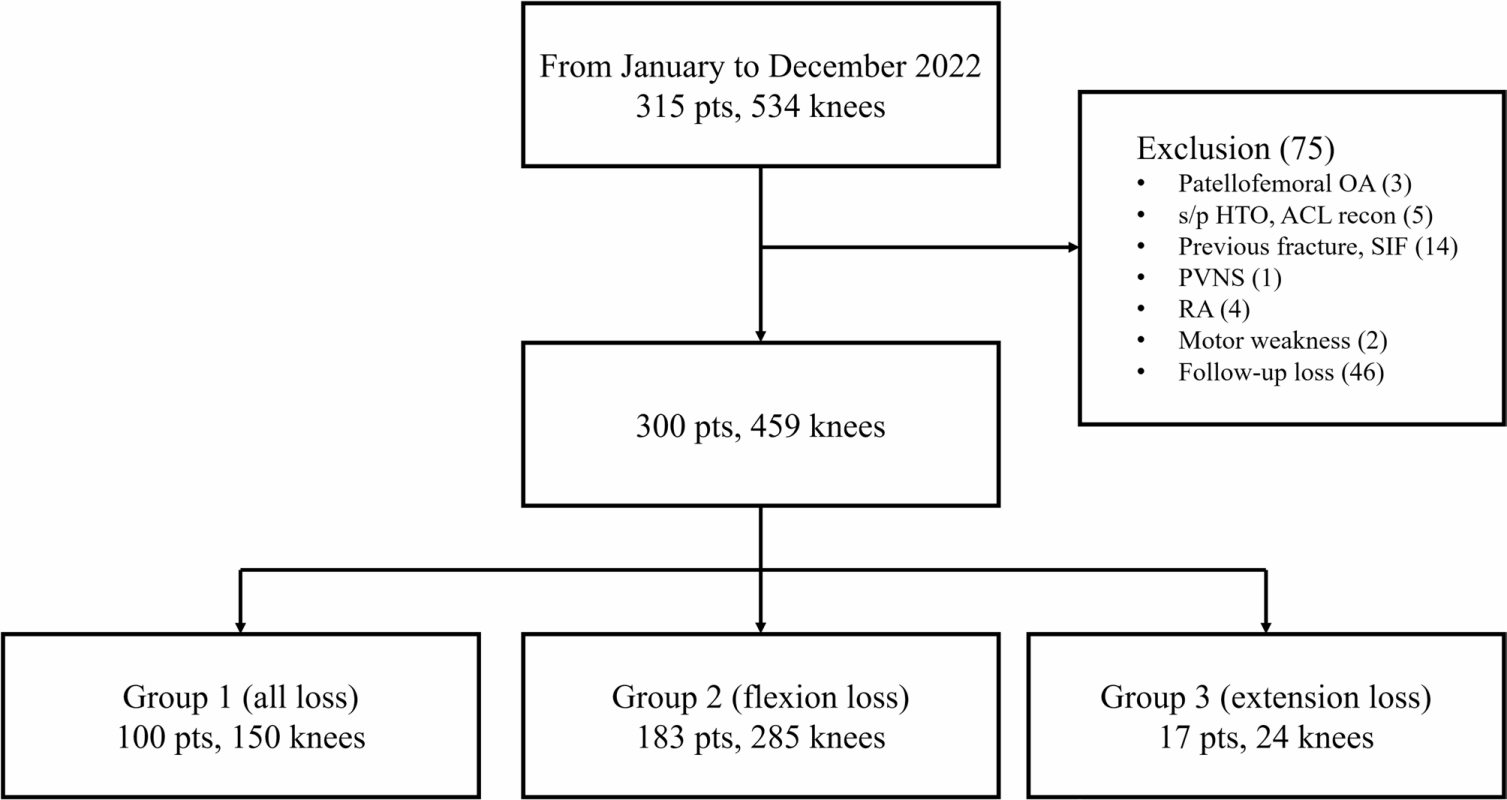
Patient selection flowchart. OA, osteoarthritis; HTO, high tibial osteotomy; ACL, anterior cruciate ligament; SIF, subchondral insufficiency fracture; PVNS, pigmented villonodular synovitis; RA, rheumatoid arthritis

A total of 300 patients with 459 knees, out of the initial 351 patients with 534 knees were included in the follow-up (Fig. 3).

Baseline characteristics, radiographic measurements, and clinical findings are summarized in Table 1. Among the participants, there were 77 men (25.7%) with an average age of 72.21 ± 7.35 years, and 223 women (74.3%) with an average age of 72.75 ± 6.56 years (*p* = 0.546). 403 knees (87.8%) were involved in the medial compartment, and TKA was performed on 259 knees (64.3%). A total of 33 patients had both knees included in the study, with different subtypes in each knee. In the comparison among the three groups, group 2 (flexion loss) showed the PTS, while group 3 (extension loss) had the smallest (*p* < 0.001), suggesting structural differences across subtypes. group 1 (all loss) exhibited more varus alignment than group 2 based on the HKA angle (*p* < 0.001) and also reported higher pain (VAS) and a shorter time to TKA, indicating more advanced disease severity. And, the proportion of patients who underwent TKA was higher in group 1 (76.0%) than in group 2 (57.2%) (*p* < 0.001). Flexion contracture was greatest in Group 3 (*p* = 0.023), while full flexion was most preserved in Group 2 (*p* = 0.002). However, there were no significant differences in age, sex distribution, BMI, PCOR, or history of partial meniscectomy among the three groups.

**Table 1:** Comparison among the subtypes of end-stage KOA

	Total	Group 1 All loss (150)	Group 2 Flexion loss (285)	Group 3 Extension loss (24)	*p*-value	Post-hoc test results
Sex (women’s knees)	349/459 (76.0%)	115/150 (76.7%)	220/285 (77.2%)	14/24 (58.3%)	0.112	
Age	72.8 ± 6.7 (72.2–73.4)	72.4 ± 6.8 (71.3–73.5)	73.3 ± 6.4 (72.6–74.0)	70.2 ± 8.8 (66-5-73.9)	0.053	
BMI (kg/m^2^)	25.7 ± 3.5 (25-4-26.0)	26.6 ± 3.7 (26.3–26.9)	25.6 ± 3.5 (25.3–25.9))	26.5 ± 3.5 (26.2–26.8)	0.284	
PTS (°)	9.9 ± 3.7 (9.6–10.3)	8.1 ± 3.3 (7.6–8.6)	11.3 ± 3.3 (10.9–11.7)	5.4 ± 2.7 (4.3–6.6)	< 0.001	2 > 1 > 3
PCOR (°)	0.5 ± 0.1 (0.5–0.5))	0.5 ± 0.1 (0.4–0.5)	0.5 ± 0.0 (0.5–0.5)	0.5 ± 0.0 (0.4–0.5)	0.428	
HKA (°)	7.6 ± 5.9 (7.0-8.1)	9.8 ± 7.0 (8.6–11.0)	6.3 ± 5.0 (5.7-7.0)	7.8 ± 5.9 (6.1–9.6)	< 0.001	1 > 2
VAS	5.2 ± 2.6 (5.0-5.4)	6.3 ± 2.4 (5.9–6.7)	4.6 ± 2.5 (4.3–4.9)	5.4 ± 2.2 (4.4–6.3)	< 0.001	1 > 2
Medial compartment involvement*	403/459 (87.8%)	145/150 (96.7%)	235/285 (82.5%)	23/24 (95.8%)	< 0.001	2 > 1
Time to surgery (months)	9.6 ± 8.6 (8.8–10.3)	7.1 ± 7.7 (5.9–8.4)	11.0 ± 8.7 (10.0–12.0)	7.5 ± 7.6 (4.2–10.7)	< 0.001	2 > 1
Flexion contracture (°)	6.2 ± 6.5 (5.2–7.1)	6.9 ± 7.2 (5.0-8.7)	5.3 ± 5.4 (4.4–6.3)	10.00 ± 9.6 (4.5–15.5)	0.023	3 > 2
Full flexion angle (°)	118.7 ± 12.7 (116.9-120.5)	114.3 ± 13.4 (110.8-117.7)	121.2 ± 11.9 (119.0-123.4)	117.5 ± 11.2 (111.0-124.0)	0.002	2 > 1
TKA (%)	295/459 (64.3%)	114 (76.0%)	163 (57.2%)	18 (75.0%)	< 0.001	1 > 2
Meniscectomy history (%)	39/459 (8.5%)	12 (8.0%)	12 (9.1%)	1 (4.2%)	0.680	
Follow-up duration (months)	13.5 ± 6.2 (12.9–14.1)	13.3 ± 5.9 (12.3–14.2)	13.8 ± 6.4 (13.1–14.6)	12.1 ± 6.0 (8.6–13.6)	0.105	

All quantitative variables are presented as mean ± standard deviation (95% CI) or percentage

*KOA* knee osteoarthritis, *BMI* body mass index, *PTS* posterior tibial slope, *PCOR* posterior condylar offset ratio, *HKA* hip-knee-ankle angle, *VAS* visual analogue scale, *TKA* total knee arthroplasty, *CI* confidence interval

*Statistical comparison involving Group 3 may be underpowered due to the small number of events

In comparing intraoperative measurements among the three groups, the extension gap was largest in group 2 (13.2 ± 2.2), and the flexion gap was largest in group 3 (15.4 ± 2.6); however, there were no statistically significant differences (Table 2).

**Table 2:** Comparison of intra-operative measurements among the groups

	Group 1 All loss	Group 2 Flexion loss	Group 3 Extension loss	*p*-value
Extension gap (mm)	12.6 ± 2.7	13.2 ± 2.2	13.0 ± 1.6	0.246
Extension angle (°)	3.7 ± 2.5	3.0 ± 2.3	2.5 ± 2.6	0.200
Flexion gap (mm)	14.4 ± 2.2	14.6 ± 2.5	15.4 ± 2.6	0.376
Flexion angle (°)	6.5 ± 3.4	6.6 ± 4.2	7.4 ± 3.1	0.728

All quantitative variables are presented as mean ± standard deviation or percentage

**Fig. 4:**
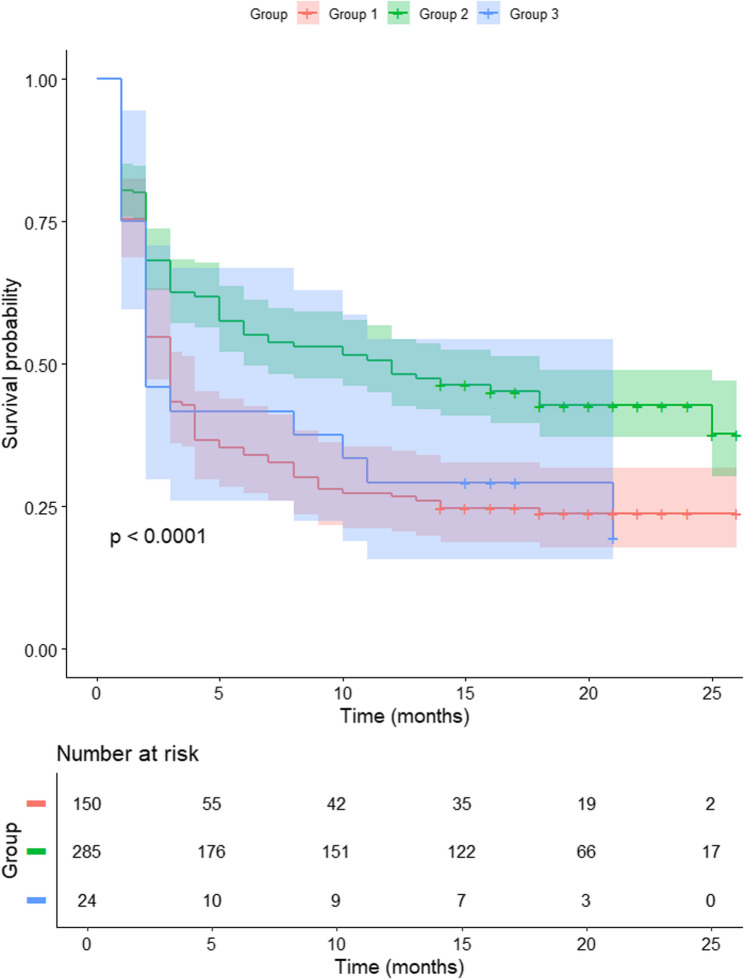
Kaplan–Meier survival analysis with total knee arthroplasty (TKA) as the endpoint

As of January 2024, the 1-year survival rate was 48.1% for group 2, 29.2% for group 3 and 26.7% for group 1 (log-rank test, *p* < 0.001). The survival rate of group 2 was statistically higher than that of group 1 and group 3 (*p* < 0.001 and *p* = 0.044, respectively), while there was no statistical difference in survival rates between group 1 and group 3 (*p* = 0.934) (Fig. 4).

## Discussion

The most important finding of this study was that even in end-stage OA, radiographic and clinical differences were observed depending on the pattern of joint space loss in different positions. Patients with joint space loss in the knee extension position exhibited features of “more severe OA,” with group 1, which also had joint space loss in flexion, showing the most severe characteristics. Although groups 2 and 3 did not show statistically significant differences in most parameters, group 3, which exhibited joint space loss only in extension, showed a more severe pattern compared to group 2.

Knee joint space is maintained by the articular cartilage and meniscus [12]. Therefore, narrowing of the joint space observed in simple radiographs indicates damage to these structures within the joint. During knee flexion, the contact point between the femoral condyle and tibial plateau moves posteriorly [13]. As a result, the joint space visible in SEV reflects the anterior part of the tibial plateau, while the joint space in FFV reflects the posterior part. Thus, based on the results of our study, KOA with different subtypes is likely to exhibit different patterns of joint damage.

It is unsurprising that group 1, which lacked joint space sparing in both SEV and FFV, showed more severe features of OA—such as greater deformity [14, 15], increased pain, and reduced range of motion [16] —compared to the other groups with joint space sparing. In advanced KOA, it is clear that any spared joint space will inevitably be lost over time. However, the difference in PTS according to the joint space loss pattern is noteworthy. Generally, a larger PTS is known to increase anterior shearing force and posterior compression force, thereby elevating the risk of anterior cruciate ligament (ACL) rupture [17] and meniscus root tear [18], among other negative effects. However, and interestingly, in advanced KOA, a larger PTS may be associated with reduced symptom severity. In our study, group 2 exhibited a larger PTS compared to the other groups, suggesting that knees with a larger PTS are more likely to have joint space sparing during extension. Several reports support our finding. First, an increased PTS leads to greater anterior shearing forces [19] and redistribution of peak stress locations on the tibial plateau. Rodner et al. reported that in ACL-deficient knees, an increase in PTS shifts the location of peak contact stress posteriorly [20]. Given that many patients in our study were at KL grade 4, some of them may have had ACL deficiency or insufficiency [21, 22]. Consequently, knees with a larger PTS are likely to exhibit greater damage to the posterior part of the tibial plateau while preserving the anterior part. Second, an increased PTS might reduce peak stress in the tibiofemoral joint, potentially leading to less joint destruction. According to Lee et al., in PS type TKA, an increase in PTS up to 10 degrees is associated with a reduction in contact stress [23]. Although this finding is based on prosthetic knees rather than natural knees, it suggests a potential protective effect of increased PTS. In our study, the larger PTS observed in group 2 may have been associated with less joint damage. These findings are consistent with the observation that knees with a larger PTS tended to exhibit joint space preservation in SEV. This may explain why group 3, with a smaller PTS, tended to show joint space loss in SEV, possibly reflecting earlier anterior compartment degeneration.

Group 2, characterized by joint space sparing during extension, reported less pain than the other groups, which may be attributed to the different functional demands of knee extension and flexion. In daily life, weight-bearing occurs primarily during standing or walking, essential for maintaining normal activities. Typically, when standing, the knee is extended, and during walking, the stance phase involves keeping the knee flexed to less than 30 degrees [24]. Therefore, an extended knee plays a crucial role in weight bearing during normal activities. Kumar et al. reported that loading is highest in both the medial and lateral compartments during the early stance phase immediately after heel strike [25], which also occurs when the knee is nearly fully extended. Thus, if joint space is lost and there are no structures to absorb shock in an extended knee, it is likely to cause significant pain. In contrast, a knee flexed beyond 45 degrees is more relevant for activities such as stair climbing, squatting, or kneeling. These activities are less critical for daily life compared to standing and walking, and can be avoided more easily. Therefore, group 2, which showed joint space loss only in the flexed position, likely experienced less frequent pain compared to the other groups and thus may take longer to reach the point of requiring surgery. This was clearly demonstrated in our survival analysis, which showed that Group 2, with joint space sparing in extension, exhibited higher survival compared to the other groups without such sparing (Fig. 4).

In our study, there were no statistical differences in intra-operative extension and flexion gaps among the three groups. This lack of differences may be due to the fact that intra-operative gaps were measured after performing distal femur and proximal tibia bone cuts. In group 2, where the joint space was larger during extension, it is likely that some cartilage remained in the anterior part [13]. Consequently, when bone cuts are made using a stylus, the actual amount of bone removed in group 2 may be less than in the other groups, which has no remaining cartilage, leading to a smaller extension gap. Conversely, applying the stylus to the midpoint of the tibial plateau in knees with a large PTS may result in relatively more bone resection in the anterior part, potentially increasing the extension gap. Due to the combined influence of these factors, the extension gap did not show a significant difference. Furthermore, despite the known association of larger PTS with a greater flexion gap [26], our study found no differences in the flexion gap among the groups. Since the target slope angle was the same, in knees with a larger PTS, the amount of bone resection in the posterior part was reduced during bone cutting, resulting in no significant difference in the gap compared to the other groups.

During the study design, we hypothesized that the condition of the meniscus or PCOR might affect end-stage KOA subtypes. However, this was not found to be the case in our study. PCOR is known to potentially impact both flexion and extension gaps [27, 28], but it was not associated with the subtype of KOA. Similarly, while joint space narrowing is associated with meniscal conditions such as tears or extrusion [29], and partial meniscectomy is known to accelerate joint space narrowing [30], a history of partial meniscectomy was not related to the pattern of joint space loss. However, since this was based solely on patient history without a quantitative assessment of the meniscus condition, further research is needed to explore this aspect more thoroughly.

Our study had several limitations. First, since this was a retrospective study with a relatively small sample size—particularly in group 3—and was conducted at a single institution, selection bias could not be completely eliminated. Second, the time to surgery and indications for deciding on surgery may vary between institutions, which could result in different percentages compared to our findings. However, since all surgeries were decided by the patients, intentional bias in specific groups is unlikely. Third, as the analysis included both knees from some patients, statistical dependency may have existed. Although no formal adjustment was made, 33 out of 159 patients with bilateral knees (20.8%) exhibited different subtypes between sides, indicating that the classification was not completely dependent within individuals. Fourth, as with all studies based on plain radiographs, the potential for technical variability is inherently present, and patient conditions such as severe flexion contracture may have influenced the radiographic assessments. Fifth, while our study was able to identify associations between KOA subtypes and anatomical structures, causality could not be determined. Sun et al. reported that PTS is larger in elderly patients [31], and Chiu et al. found that PTS is larger in OA knees compared to non-OA knees [32]. Thus, it appears that as arthritis progresses with age, PTS increases, which may influence the subtype of end-stage OA. However, additional longitudinal or biomechanical research is needed to clarify these causal relationships.

Nevertheless, our findings may help clinicians better understand patients with end-stage KOA. By identifying the pattern of joint space loss, clinicians may more accurately predict symptom severity in specific functional postures and personalize treatment plans accordingly, including patient education, activity modification, targeted physical therapy (e.g., focusing on range of motion vs. load-reducing strategies), and optimal timing of surgery. Furthermore, as our study demonstrated differences in time to TKA between groups, this classification may also assist in surgical planning and counseling by identifying patients who are likely to benefit from prolonged conservative management versus those who may require earlier surgical intervention.

## Conclusion

We proposed a novel classification of end-stage knee osteoarthritis based on joint space loss patterns in extension and flexion views. The three subtypes showed distinct clinical and radiological features, with group 2 (flexion loss only) demonstrating milder symptoms and longer time to surgery. This classification may assist in understanding symptom patterns and planning patient-specific treatment. Additionally, our findings suggest that a larger PTS may be associated with less symptom severity in advanced KOA, which contrasts with its traditionally recognized biomechanical disadvantages. Further studies are needed to validate its clinical utility.

## Supplementary Information

Supplementary Material 1.

## Data Availability

The data sets used and/or analyzed in the current study are available from the corresponding author on reasonable request.
