# Supplementary material for: Clinical and radiological characteristics of novel subtypes of end-stage knee osteoarthritis based on joint space loss patterns in standing extended view and fixed flexion view
Source: BMC Musculoskelet Disord. 2025 Jul 22;26:696. doi: 10.1186/s12891-025-08943-y (PMC12281949; doi:10.1186/s12891-025-08943-y)
Supplement: Supplementary file 1 — Supplementary Material 1. [file 12891_2025_8943_MOESM1_ESM.docx]

**Table 1.** Comparison among the subtypes of end-stage KOA (including post-hoc p-values)

|  | Total | Group 1  All loss (150) | Group 2  Flexion loss (285) | Group 3  Extension loss (24) | p-value | Post-hoc test results |
| --- | --- | --- | --- | --- | --- | --- |
| Sex (women’s knees) | 349/459 (76.0%) | 115 (76.7%) | 220 (77.2%) | 14 (58.3%) | 0.112 |  |
| Age | 72.8 ± 6.7 | 72.4 ± 6.8 | 73.3 ± 6.4 | 70.2 ± 8.8 | 0.053 |  |
| BMI (kg/m^2^) | 25.7 ± 3.5 | 26.6 ± 3.7 | 25.6 ± 3.5 | 26.5 ± 3.5 | 0.284 |  |
| PTS (°) | 9.9 ± 3.7 | 8.1 ± 3.3 | 11.3 ± 3.3 | 5.4 ± 2.7 | **<0.001** | **2 > 1 (p<0.001)**  **1> 3 (p<0.001)** |
| PCOR (°) | 0.5 ± 0.1 | 0.5 ± 0.1 | 0.5 ± 0.0 | 0.5 ± 0.0 | 0.428 |  |
| HKA (°) | 7.6 ± 5.9 | 9.8 ± 7.0 | 6.3 ± 5.0 | 7.8 ± 5.9 | **<0.001** | **1 > 2 (p<0.001)** |
| VAS | 5.2 ± 2.6 | 6.3 ± 2.4 | 4.6 ± 2.5 | 5.4 ± 2.2 | **<0.001** | **1 > 2 (p<0.001)** |
| Medial compartment involvement* | 403/459 (87.8%) | 145/150 (96.7%) | 235/285 (82.5%) | 23/24 (95.8%) | **<0.001** | **2 > 1 (p<0.001)** |
| Time to surgery (months) | 9.6 ± 8.6 | 7.1 ± 7.7 | 11.0 ± 8.7 | 7.5 ± 7.6 | **<0.001** | **2 > 1 (p<0.001)** |
| Flexion contracture (°) | 6.2 ± 6.5 | 6.9 ± 7.2 | 5.3 ± 5.4 | 10.00 ± 9.6 | **0.023** | **3 > 2 (p=0.032)** |
| Full flexion angle (°) | 118.7 ± 12.7 | 114.3 ± 13.4 | 121.2 ± 11.9 | 117.5 ± 11.2 | **0.002** | **2 > 1 (p=0.001)** |
| TKA (%) | 295/459 (64.3%) | 114 (76.0%) | 163 (57.2%) | 18 (75.0%) | **<0.001** | **1 > 2 (p<0.001)** |
| Meniscectomy history (%) | 39/459 (8.5%) | 12 (8.0%) | 12 (9.1%) | 1 (4.2%) | 0.680 |  |
| Follow-up duration (months) | 13.5 ± 6.2 | 13.3 ± 5.9 | 13.8 ± 6.4 | 12.1 ± 6.0 | 0.105 |  |

All quantitative variables are presented as mean ± standard deviation or percentage.

*Statistical comparison involving Group 3 may be underpowered due to the small number of events.

KOA, knee osteoarthritis; BMI, body mass index; PTS, posterior tibial slope; PCOR, posterior condylar offset ratio; HKA, hip-knee-ankle angle; VAS, visual analogue scale; TKA, total knee arthroplasty.
